# Obesity-Related Knee Osteoarthritis—Current Concepts

**DOI:** 10.3390/life13081650

**Published:** 2023-07-28

**Authors:** Russka Shumnalieva, Georgi Kotov, Simeon Monov

**Affiliations:** Clinic of Rheumatology, Department of Rheumatology, Medical University of Sofia, 1431 Sofia, Bulgaria; rshumnalieva@yahoo.com (R.S.); doctor_monov@yahoo.com (S.M.)

**Keywords:** knee osteoarthritis, metabolic syndrome, obesity, pathogenesis, adipokines, disease-modifying treatment, personalized medicine

## Abstract

The knee is the joint most frequently involved in osteoarthritis and represents a significant contributor to patient morbidity and impaired functional status. Major risk factors include genetics, age, sex, mechanical load and obesity/metabolic syndrome. Recent studies highlighted the role of obesity and metabolic syndrome in the pathogenesis of knee osteoarthritis not simply through increased mechanical loading but the systemic effects of obesity-induced inflammation. The current concept of knee osteoarthritis is that of a ‘whole joint disease’, which highlights the involvement not only of articular cartilage but also the synovium, subchondral bone, ligaments and muscles. Obesity and metabolic syndrome are associated with higher levels of pro-inflammatory cytokines, increased production of adipokines with both protective and destructive effects on articular cartilage, an up-regulation of proteolytic enzymes such as matrix metalloproteinases and aggrecanases and an increase in free fatty acids and reactive oxygen species induced by dyslipidemia. These findings underscore that the adequate management of knee osteoarthritis needs to include an optimization of body weight and a beneficial mobility regimen. The possible introduction of pharmacological therapy targeting specific molecules involved in the pathogenesis of obesity-related osteoarthritis will likely also be considered in future therapeutic strategies, including personalized treatment approaches.

## 1. Introduction

Osteoarthritis (OA) is among the most common joint disorders worldwide and a significant contributor to patient morbidity and impaired functional status, leading to major socioeconomic burden [1,2,3]. The prevalence of OA worldwide over the period 1990–2019 increased by a striking 114% [4]. These numbers, however, vary widely, mainly owing to the definition used (clinical vs. radiographic OA), the population sampled and the affected joints [1]. The knee is the joint most frequently involved in OA, followed by interphalangeal joints of the hand and the hip [5]. Risk factors include genetics, age, sex, mechanical load, obesity/metabolic syndrome and others. Obesity-related OA, also more broadly described as metabolic-syndrome-associated OA, is a complex condition that not only stems from the increased mechanical load on weight-bearing joints (such as the knee and hip) but the systemic effects of obesity-induced inflammation that further increase the risk of OA in other areas (such as the hand) [2,3]. Recently, advances in our understanding of the role of metabolic syndrome in the development and progression of knee OA highlighted the importance of further intervention studies that target different components of metabolic syndrome in order to investigate whether their modification could prevent the occurrence or progression of structural damage [6]. This review aimed to present the current concepts of knee osteoarthritis pathogenesis with particular focus on the role of obesity and other components of metabolic syndrome in disease initiation and progression.

## 2. Epidemiology and Pathogenesis of Knee Osteoarthritis

Knee OA is a progressive multifactorial disease associated with chronic pain, reduced mobility and increased patient morbidity [5]. Almost four fifths of the total OA burden worldwide is attributed to knee OA, and this burden increases with obesity and age [7,8]. Incidence and prevalence vary substantially among countries due to the impact of geographical location, development, income and other socioeconomic factors, and were estimated to be around 86.7 million worldwide and around 654.1 million worldwide in 2020, respectively [8]. An analysis of OA distribution among regions based on geography and income (assessed through the sustainable development index—SDI) over the period 1990–2019 revealed that the knee was the predominantly affected joint in all geographical regions apart from high-income North America and Eastern Europe, where it was superseded by hand OA [4]. In addition, prevalence of radiographic knee OA was higher than that of symptomatic or self-reported cases [8]. Females are more commonly affected, with prevalence ranging from 19% to 24.5% (mean 21.7%), while in males, it falls within the range 10.2–13.8% (mean 11.9%). Clinical OA is defined based on history and examination and can be classified according to various criteria, the most recognized of which are those of the American College of Rheumatology (ACR), which consider the presence of crepitus, morning stiffness and bone enlargement [1]. Radiographic OA is usually assessed based on the Kellgren and Lawrence score, which takes into consideration joint space loss and the presence of osteophytes, sclerosis and cysts and grades disease severity on a scale from 0 to 4 (Table 1) [1,9].

While historically knee OA has been considered a disease primarily involving structural modifications to the joint cartilage and the subchondral bone, the synovial membrane, ligaments, muscles and Hoffa’s fat pad are also affected, thus leading to the concept of OA as a ‘whole joint disease’ [10,11].

Articular cartilage is composed of connective tissue cells known as chondrocytes situated in an extracellular matrix (ECM) built of water (more than 70%) and organic components including collagen type II, aggrecan, decorin, fibromodulin, glycosaminoglycans (chondroitin sulfate and keratan sulfate) and glycoproteins. Generally, the structural elements of the ECM are organized in the following manner: proteoglycan aggregates containing chondroitin sulfate and keratan sulfate bound to aggrecan as the ‘core protein’, which is connected to a backbone of hyaluronic acid, along with other ECM components are entrapped in a network formed by collagen type II fibrils [11,12]. All these components are produced by chondrocytes and this process is finely regulated through the action of proteolytic enzymes and is further regulated by mechanical loading through membrane-bound mechanoreceptors [11,13]. Changes in the cartilage ECM are among the first manifestations of OA. These include the cleavage of aggrecan from the hyaluronic acid backbone by proteinases of the ADAMTS family, particularly ADAMTS-4 and ADAMTS-5, as well as the disruption of the collagen network by matrix metalloproteinases (MMP), mainly MMP-13, which has a distinct avidity for collagen type II [14,15]. Proliferation and hypertrophy of chondrocytes aimed at restoring the cartilaginous matrix instead lead to release of pro-inflammatory mediators such as tumor necrosis factor-alpha (TNFα), interleukin 1β (IL-1β) and interleukin 6 (IL-6), which accelerate the degradation of the matrix and may activate the adjacent synovium [11,16].

Apart from articular cartilage, other structures of the knee joint are also affected in OA. Subchondral bone alterations are critical in the pathogenesis of OA and differ in early and late-stage OA [17]. Early changes are characterized by a deterioration of the subchondral plate, with compromised subchondral trabeculae, increased porosity and decreased bone density. On the contrary, subchondral sclerosis, increased trabecular thickness and decreased trabecular separation are all features of late-stage OA [17,18]. Two major histopathological alterations found in OA joints include bone marrow lesions, which correlate with knee OA severity [19] and subchondral bone cysts, which are usually found in weight-bearing regions of the joint and show a multitude of osteoblasts, osteoclasts and osteoprogenitor cells, suggesting a high degree of bone turnover [20]. Moreover, these cellular types show their own alterations in the knees of OA patients. For instance, osteoblasts in the OA subchondral bone have higher alkaline phosphatase activity and produce higher levels of insulin-like growth factor 1 (IGF1), transforming growth factor β1 (TGFβ1), receptor activator of nuclear factor kappa beta-ligand (RANKL) and vascular endothelial growth factor (VEGF) compared to normal osteoblasts [18].

Synovitis is a common finding in knee OA, which can be present in early as well as more advanced stages and is associated with pain, functional impairment and structural progression [11,21]. Multiple inflammatory mediators have been found in the synovial fluid of OA patients, including C-reactive protein (CRP), TNFα, IL-1β, IL-6, TGFβ1, VEGF, various fibroblast growth factors (FGFs), leukotrienes, prostaglandins and others [21,22]. In addition, synovial inflammation leads to influx of macrophages, T- and B-lymphocytes and mast cells [23]. The number of these cells and the expression of cytokines, however, have been described as moderate and lower than those in rheumatoid arthritis (RA) [23]. Interestingly, synovial inflammation induced by cartilage damage drives structural damages further in a vicious cycle by an increased production of proteolytic enzymes [24].

## 3. Risk Factors

Generally, risk factors for the development of OA can be divided into person-level factors and joint-level factors (Table 2) [1].

Old age is a major risk factor that contributes to the development of knee OA in multiple ways, including oxidative stress, muscle weakening and sarcopenia, decreased mobility and impaired proprioception [1,4]. In addition, genetics are thought to play a role in approximately 40% of cases of knee OA, in particular, genes encoding the vitamin D receptor, collagen type II, IGF1 and growth differentiation factor 5 [25]. The role of dietary habits, smoking and alcohol consumption is not yet clear. As mentioned above, females are more commonly affected by knee OA than males. Females generally tend to have narrower femurs, thinner patellae, greater angle of insertion of the quadriceps tendon and smaller tibial condyles—all factors that alter knee kinematics [26]. The role of obesity/overweight and metabolic syndrome will be reviewed in detail below.

At the joint level, particular repetitive joint movements have been associated with a higher risk for knee OA. Prolonged and frequent squatting predisposes elderly individuals to tibiofemoral knee OA. Moreover, occupational activities involving squatting or kneeling more than two hours daily were associated with two-fold significantly increased risk of moderate to severe radiographic knee OA [27]. Injuries and tears to the ligamentous apparatus of the joint, including meniscal lesions, lead to decreased resistance to mechanical forces, including shear stress, tension and compression [11]. Rupture of the anterior cruciate ligament (ACL) in particular causes joint instability and is responsible for early OA developing in 10 to 15 years in 13% of cases. When such injuries are combined with damage to collateral ligaments, menisci, cartilage and/or subchondral bone, this percentage may rise to as high as 40% [28].

## 4. Obesity, Metabolic Syndrome and Knee OA

Overweight (defined as a body mass index (BMI) > 25 kg/m^2^) and obesity (defined as a BMI > 30 kg/m^2^) have long been recognized as major risk factors for the development of OA in general and knee OA in particular [2,3,29]. Higher BMI levels correlate with higher pain scores, diminished physical activity and disability [3]. In a broader aspect, metabolic syndrome, a heterogeneous disorder characterized by central obesity, hypertension, dyslipidemia and impaired fasting glucose or diabetes has also been associated with knee OA [6,30,31,32]. As evidenced by results from the Chingford study, its components other than obesity (high blood glucose, hypertension and hypercholesterolemia) are independently associated with both unilateral and bilateral knee OA [30]. These findings have recently been challenged in studies adjusting for BMI as continuous variable, which found that other components of metabolic syndrome may increase the risk of incident knee OA but not independent of BMI and body weight [32]. Hypertension and elevated diastolic blood pressure in particular were found to be associated with symptomatic knee OA, but these results are not unequivocal and establishing a causal relationship is further hampered by the fact that patients with symptomatic knee OA often take non-steroidal anti-inflammatory drugs (NSAIDs) that can raise blood pressure on their own [32].

The mechanisms by which metabolic factors influence the development of OA are still under investigation. It is well known that overweight and obesity increase the risk of OA by increasing the mechanical load on lower extremity joints but it has been shown that they are also responsible for higher incidence of hand OA, thus underscoring their systemic effects which merit further research [3]. The moderate loading of knee joints as part of routine daily activities in an important factor for the maintenance of articular cartilage homeostasis through the activation of mechanoreceptors. However, abnormal excessive mechanical load leads to disruption of cartilage homeostasis and deformation of normal joint morphology, which induces and accelerates the progression of knee OA [2]. One study on an experimental model of chronic compression of the knee for 20 weeks found a decrease in cartilage thickness and cellularity and more pronounced histological degeneration, paired with increased subchondral bone thickness [33]. Another in vivo model of cyclic knee loading for 2 weeks was subjected to study by magnetic resonance imaging (MRI), which revealed the presence of bone marrow lesions, bone resorption and increased tissue microdamage [34]. The anatomical configuration of the knee (a hinge joint) as opposed to the hip (a ball-and-socket joint) means that it relies heavily on the adjacent capsule, ligaments and tissues for maintaining stability and protection against shear stress, compressive and axial-loading forces; any damage to these structures may cause abnormal joint loading, which provides a reason why obesity and overweight have a more significant impact on the knee than on the hip [35]. 

In addition, obese patients usually have higher thigh girth. In order to avoid thigh contact while walking, such patients develop greater hip abduction and varus malalignment of the knee, which reduces the contact area along which the mechanical stress is transferred and causes preferential load and subsequent damage of the medial aspect of the articular cartilage [2]. Furthermore, obesity leads to a relative muscle dysfunction. In obese females with knee OA, a negative correlation has been described between body weight and knee extensor strength (quadriceps) [2]. A suggested possible explanation involves an excessive infiltration of adipose cells into muscle tissue [36]. On the other hand, the combination of obesity and sarcopenia (often seen in elderly individuals due to physical dysfunction) is in itself a risk factor for development of knee OA, as evidenced by a cross-sectional study of 2893 patients [37]. 

Yet, these mechanical factors are merely the ‘tip of the iceberg’. It has been suggested that obesity’s impact on joints and its essence as a major factor in the development of OA lies in the union between increased weight and the ‘chronic micro-inflammatory state’ [38]. Weight overloading of joints in itself may cause an increase in the levels of IL-1β and TNFα and stimulate the degradation of cartilage ECM [39]. However, another key player is adipose tissue and its role as a potent source of cytokines, chemokines and other biologically active mediators, collectively known as adipokines [40]. Some of these molecules maintain a chronic state of low-grade inflammation in obese individuals (such as TNFα, IL-1β, IL-6, adiponectin and leptin) and promote a catabolic state that degrades the joint, while others help preserve cartilage integrity [39,40]. In addition, the cellular composition of adipose tissue in thin and obese individuals varies. Obesity is associated with a change in phenotype from M2 macrophages to classic M1-activated macrophages that release pro-inflammatory cytokines [41]. These complex mechanisms further involve other pathological conditions associated with obesity in the context of metabolic syndrome, such as dyslipidemia and insulin resistance [39,42].

Recent studies reported a link between the lipotoxicity present in dyslipidemia and the development of OA [43,44]. One of the aberrant responses seen in chondrocytes over the course of OA is the accumulation of intracellular lipids, the levels of which correlate with disease severity [39]. Furthermore, two enzymes important in lipid metabolism, namely cholesterol 25-hydroxylase and 25-hydroxycholesterol 7α-hydroxylase, were found to increase in OA states and were associated with a decrease in anabolic factors and the development of key OA features such as formation of osteophytes, synovitis and subchondral sclerosis. The impact of these elevated enzymes involved a higher expression of MMP and members of the ADAMTS family [45]. In addition, dyslipidemia leads to an increase in free fatty acids and reactive oxygen species (ROS), which play a role in cartilage degradation [46,47]. These substances stimulate the release of pro-inflammatory cytokines either directly or through the activation of resident macrophages and cause oxidative stress by inducing mitochondrial dysfunction in chondrocytes [48]. High levels of total cholesterol and triglycerides, as well as decreased high-density lipoprotein (HDL) are also associated with knee structural damage in OA and the occurrence of bone marrow lesions [48,49]. On the other hand, high levels of low-density lipoprotein (LDL) provoke synovial inflammation and ectopic bone formation. The role of LDL in the development and progression of OA may also be explained by the stimulation of chondrocytes and synovial cells [43]. In line with the above, several studies showed a decreased risk of OA incidence and knee pain in patients taking statins as cholesterol-lowering medication [50,51]. However, a recent report on a population-based cohort of 255 participants with knee pain and pre-radiographic OA showed no benefit of statin therapy on the incidence and progression of cartilage damage, formation of osteophytes and bone marrow lesions as assessed through MRI, suggesting that statins do not prevent the progression of knee OA [52].

Insulin resistance was also implicated in knee OA in the context of obesity and metabolic syndrome. Insulin has an important anabolic impact on articular cartilage by increasing the synthesis of collagen type II and proteoglycans, promoting chondrocyte proliferation and inhibiting the action of aggrecanases, TNFα and IL-1β [39,53]. Insulin resistance, therefore, likely makes the synovium and articular cartilage more susceptible to the pro-inflammatory activity of the above mediators. Furthermore, insulin resistance leads to elevated levels of free fatty acids, which, as described above, are directly involved in cartilage degradation [46,47,54]. While insulin stimulates chondrocyte proliferation, one study found that its elevated serum levels (i.e., hyperinsulinemia) seen in insulin resistance, in fact, inhibited chondrocyte differentiation and maturation [55]. One study on an experimental mouse model of OA investigated the effect of metformin, a potent first-line treatment used to increase insulin sensitivity in patients with insulin resistance, diabetes and obesity, as a possible disease-modifying agent in OA, and found that the combination of metformin and alendronate, a bisphosphonate with anti-resorptive properties used in the treatment of osteoporosis, led to a decrease in the degree of cartilage degradation [56].

The role of adipokines in the development of OA continues to be meticulously researched. Contrasting results have been reported on the role of adiponectin. Increased levels of circulating adiponectin were recently correlated with a higher radiographic score in knee OA, particularly with joint space narrowing and the formation of osteophytes [57]. On the other hand, it has been hypothesized that adiponectin plays a protective role, as it tilts the balance from M1 to M2 macrophages in adipose tissue which have anti-inflammatory properties. In addition, it appeared to decrease the levels of TNFα [58]. By regulating lipid metabolism in obese patients, adiponectin reduces the levels of free fatty acids and triglycerides in the serum, thereby reducing certain risk factors for obesity-related knee OA [59]. Adiponectin may also exert cartilage protection by increasing the expression of the tissue inhibitor of MMP-2 (TIMP-2) and by decreasing IL-1β-stimulated expression of MMP-13 [59]. TIMP-2 in itself inhibits the action of MMP and ADAMTS [60].

Leptin, another major adipokine, is a peptide hormone, the levels of which are significantly increased in obesity [61]. Chondrocytes from osteoarthritic cartilage express higher levels of the leptin gene; furthermore, the leptin gene and its receptor gene show an association with OA as evidenced by single nucleotide polymorphism analysis [61]. Interestingly, leptin shows a stronger association with knee OA in females than males [62]. One reason behind this discrepancy could be that knee OA in males is more biomechanical in nature, while systemic low grade inflammation is more at play in the development of female knee OA [63]. In addition, serum levels of leptin correlate with a higher BMI, a higher Western Ontario and McMaster Universities Arthritis Index (WOMAC) pain score and higher radiographic score in knee OA [64]. Increased serum leptin accelerates the process of joint inflammation in OA, while its decrease in weight loss improves symptoms in OA patients [61]. On the other hand, locally increased leptin inside OA-affected joints correlates with BMI and may even be higher than that in the serum [65]. Leptin stimulates the production of pro-inflammatory cytokines such as IL-1β, IL-6, vascular cell adhesion molecule-1 (VCAM-1), and prostaglandin E2 by articular chondrocytes [66]. It also upregulates the synthesis of proteolytic enzymes such as MMP-1, MMP-3, MMP-9 and MMP-13 by chondrocytes [67]. 

Another adipokine, resistin, appears to be important in the development of insulin resistance in patients with diabetes and OA [68]. Resistin inhibits insulin-induced glucose uptake and increases the expression of MMP and VEGF receptors [69,70]. It has been associated with the progression and severity of knee OA, structural damage and serum leptin levels and was found to stimulate the production of pro-inflammatory cytokines and MMP by chondrocytes [71,72]. Serum levels of resistin were shown to be higher in patients with OA and they may inhibit the synthesis of cartilage ECM. Moreover, the serum levels of resistin correlate with the occurrence of bone marrow lesions and cartilage defects [72]. Visfatin, a pro-inflammatory adipokine that stimulates the expression of TNFα, IL-1β, IL-6, MMP and VEGF, has also been implicated in the pathogenesis of OA. By promoting the activity of ADAMTS4 and ADAMTS5, as well as MMP-3 and MMP-13, and decreasing the synthesis of collagen type II and high molecular weight proteoglycans, it has a profound catabolic effect on articular cartilage. Its levels in serum and synovial fluid were shown to correlate with radiographic damage, OA activity, levels of CRP and degradation markers of aggrecan and collagen type II [73]. Vaspin, on the other hand, appears to be an adipokine with anti-inflammatory properties. It counters the effects of ROS, TNFα and pro-inflammatory adipokines such as leptin and resistin [74]. It is a mediator expressed by cartilage, menisci, synovial tissue and the infrapatellar fat pad, but its expression in knee OA is severely diminished and only observed in the superficial cartilage zone [73]. Serum concentrations of vaspin in healthy individuals are higher than those in OA patients. In addition, in patients with knee OA, vaspin levels in the synovial fluid are lower than those in the serum, all of which suggest a protective role for this adipokine in OA.

## 5. Sarcopenia and Obesity-Related Knee OA

As mentioned above, muscle dysfunction is also a major element in the development of knee OA. Sarcopenic obesity, a separate body composition entity, describes cases where the increase in body weight caused by obesity is offset by a decrease in muscle mass [37]. It is the result of a plethora of factors, including changes in physical activity, protein intake and altered levels of hormones such as parathyroid hormone, insulin, sex hormones and vitamin D and is more closely associated with knee OA than non-sarcopenic obesity [37]. Adipokines, through their mediation of a chronic low-grade inflammatory state, are, therefore, key in this progressive muscle loss; however, they also exhibit a ‘dualistic’ character. Adiponectin appears to have a protective role against the breakdown of muscle proteins and its serum levels were higher in sarcopenic patients, possibly as a compensatory mechanism. Leptin, on the other hand, showed a negative correlation with the skeletal mass index and, thus, increases the risk of sarcopenic obesity [75]. This triad of obesity, sarcopenia and osteoarthritis is not only significantly inter-related but also a key element of physical performance and, as such, a major determinant of quality of life.

Recent studies shed more light on the relationship between sarcopenia, sarcopenic obesity and OA. One longitudinal cohort study of 1653 participants without radiographic evidence of knee OA and subsequent follow-up at 60 months found a statistically significant increase in the risk of knee OA development in obese males and females, as well as females with sarcopenic obesity, but not in males with sarcopenic obesity or males and females with sarcopenia and no obesity [76]. Other studies concluded that sarcopenia alone was also associated with OA and reported that lower muscle mass of the lower limbs is correlated with knee OA [77]. As previously suggested, measuring the cross-sectional area of the quadriceps muscle may be a sensitive marker of knee OA progression [78]. In addition, an increase in the cross-sectional area of the vastus medialis was shown to be associated with reduced knee pain, reduced medial tibial cartilage loss and lower risk of total knee replacement in a longitudinal study of 117 patients over the course of 4.5 years [79]. A recent meta-analysis found the prevalence of sarcopenia in knee OA to be as high as 45.2% and two times higher than that in controls [80]. The authors discussed that quadriceps weakness could be related not only to sarcopenia but to an arthrogenous muscle inhibition stemming from an altered afferent input from the OA knee and a subsequent impaired efferent motor neuron stimulation of the quadriceps [80]. An interesting link has been explored between myostatin, an important regulator of muscle tissue metabolism, and knee OA [81]. Myostatin is a myokine protein of the TGFβ family, which negatively regulates skeletal muscle growth and whose deficiency suppresses the accumulation of adipose tissue. Serum and synovial fluid concentrations of myostatin were higher in patients with knee OA and correlated with a higher Kellgren–Lawrence score [81]. One way through which myostatin may drive the progression of OA is through the stimulation of osteoclastogenesis and joint destruction, since myostatin-deficient mice were found to have reduced RANKL-induced promotion of osteoclasts and a reduced number of osteoclasts [82]. Furthermore, OA synovial fibroblasts incubated with TNFα, IL-1 and IL-17 showed increased expression of myostatin [82]. On the other hand, myostatin suppression through myostatin-specific antibodies led to a decrease in the levels of TNFα and IL-6 [81], which also underscores the key relationship between muscle and cartilage tissue in the setting of OA. 

## 6. Therapeutic Interventions in Obesity-Related Knee OA

Despite the accumulating evidence on the relationship between metabolic syndrome and OA, specific recommendations for the therapeutic management of obesity-related knee OA are yet to be published [83]. Guidelines for the management of knee OA in general have been published by ACR [84] and the Osteoarthritis Research Society International (OARSI) [85] and apply to cases of obesity-related knee OA; however, one should always consider the various aspects of metabolic syndrome and comorbidities in such patients, which may lead to adverse outcomes when using particular pharmacological approaches. The abovementioned guidelines have been summarized in Table 3.

As seen from the above summary, it is strongly recommended that patients with knee OA follow dietary weight management and this is particularly true of cases of obesity-related knee OA. Moreover, symptom and functional improvement increases with the amount of weight loss. Long-term weight loss of 10–19.9% of baseline body weight had a significant effect on clinical symptoms and mechanical properties as opposed to less weight loss [86]. In fact, a loss of ≥5% of body weight is associated with improved clinical and mechanical outcome [84]. In addition, weight loss improves traditional cardiovascular risk factors and metabolic abnormalities observed in patients with obesity-related knee OA [83]. Weight loss strategies are also of particular importance in cases of established knee OA, in order to slow down disease progression [2]. At a molecular level, weight loss has been shown to correlate with a reduction in cartilage thickness loss and increased proteoglycan content in the medial knee compartment, increased serum levels of cartilage synthesis markers and decreased serum levels of cartilage degradation markers [87,88].

Aerobic exercise and muscle strengthening are widely recommended interventions in the management of knee OA. These include walking on a treadmill or supervised indoor fitness walking, as well as cycling on stationary bicycles [84]. Isometric exercise involves exercising at discrete joint angles, while dynamic exercise, which features isokinetic and isotonic training, is based on resistance training aimed at muscle strengthening [2]. One randomized control trial of 289 obese patients found that an exercise regimen aimed at strengthening the quadriceps, which was followed over a period of two years, was associated with a significant reduction in knee pain [89]. In addition, resistance training has been shown to promote muscle strength, reduce pain and improve joint mobility in 50–70% of patients with obesity-related knee OA [2]. A specialized neuromuscular training program helped achieve better proprioception and postural control, as well as muscle strength, activation and coordination [90]. Aquatic exercise is particularly useful, as it combines aerobic training and exercises aimed at improving joint range of motion in a low-impact environment [84]. Currently, there is insufficient evidence to suggest one form of exercise over another and it is important to take into consideration the patient’s preference and accessibility to a given form of exercise, which are key in their adherence to the prescribed physical activity regimen. Last but not least, devices such as tibiofemoral or patellofemoral braces and gait aids are useful in patients where knee OA is associated with a major impact on ambulation, joint stability and pain [84,85]. Foot orthotic devices have been shown to improve knee pain and stiffness and decrease the use of analgesic medications [91]. 

While oral NSAIDs and intraarticular CSs are strongly recommended in knee OA in the general population, such drugs should be avoided or, at the very least, used with particular caution in cases of obesity-related knee OA due to cardiovascular comorbidities [83]. Symptomatic slow-acting drugs such as chondroitin and glucosamine and some anti-oxidants such as ginger extracts and curcumin may be useful in these patients, although neither are recommended in the guidelines of ACR and OARSI [83]. When choosing to use NSAIDs, topical forms should be preferred over oral ones, due to an equivalent effect on knee pain with fewer adverse reactions due to slow systemic absorption [92]. Therefore, topical NSAIDs could be preferred for older patients and those with cardiovascular and gastrointestinal comorbidities, as well as patients with obesity-related knee OA [92]. Intraarticular injections of CSs and hyaluronic acid are generally considered beneficial and have few adverse effects. OARSI’s statement maintains that intraarticular injections of CSs provide short-term pain relief; however, the application of hyaluronic acid may have a better effect on pain relief over a longer period (12 weeks and beyond) and a more favorable safety profile over repeated injections of CSs [85]. ACR’s guidelines, on the other hand, include a strong recommendation for intra-articular injections of CSs but do not recommend the use of hyaluronic acid [84]. Furthermore, in obesity-related knee OA, the success rate of intraarticular injections is lower than that in patients with normal BMI [83]. A study of 166 participants with symptomatic knee OA, 42.8% of whom were obese, found that the WOMAC score 6 months after intraarticular injection of a viscosupplement decreased significantly in all subgroups; however, the score was significantly lower in non-obese vs. obese patients [93]. These results highlight that any pharmacological interventions in obesity-related knee OA would have a lower success rate unless low-calorie diet and physical activity aimed at weight loss and decreased adipose tissue deposits are maintained as the cornerstone of therapeutic management [94]. Finally, the use of antiobesity drugs may be beneficial in patients with BMI > 30 and comorbidities, but their role has not been well established in patients with concomitant OA and it is unclear whether their addition would yield beneficial results [94].

## 7. Conclusions

Knee OA is a disorder of increasing incidence and significance, which is associated with increased patient morbidity, decreased mobility and reduced quality of life. Recent findings indicated the key role of obesity and other components of metabolic syndrome such as dyslipidemia and insulin resistance on the initiation and progression of knee OA, which far exceed the boundaries of the weight overload hypothesis. Therefore, adequate management of knee OA needs to include an optimization of body weight, reduction in BMI and waist circumference and a beneficial mobility regimen. The possible introduction of pharmacological therapy targeting specific molecules involved in the pathogenesis of obesity-related OA, such as adipokines and pro-inflammatory cytokines, will likely also be considered in future therapeutic strategies, including personalized treatment approaches.

## Figures and Tables

**Table 1:** Grades of knee OA based on Kellgren and Lawrence score [9].

Grade	Description
Grade 1	Doubtful narrowing of the joint space with possible osteophyte formation.
Grade 2	Possible narrowing of the joint space with definite osteophyte formation.
Grade 3	Definite narrowing of joint space, moderate osteophyte formation, some sclerosis and possible deformity of bony ends.
Grade 4	Severe narrowing of the joint space, large osteophyte formation, with marked sclerosis and definite deformity of bone ends.

**Table 2:** Some major risk factors for the development of osteoarthritis.

Person-Level Factors	Joint-Level Factors
Genetics	Knee injury
Old age	Mechanical loading
Female gender	Repetitive joint use
Obesity/metabolic syndrome	Muscle weakness
Dietary habits	Joint laxity
Socioeconomic factors	Bone density

**Table 3:** Summary of the guidelines of ACR and OARSI on the management of knee osteoarthritis by strength of recommendation [84,85].

Type of Recommendation	ACR	OARSI
Strongly recommended	Exercise Self-efficacy and self-management programs Weight loss Cane Tibiofemoral knee brace Tai Chi Oral and topical NSAIDs Intraarticular CSs	Core recommendation—Arthritis Education; Structured Land-Based Exercise Programs (Type 1—strengthening and/or cardio and/or balance training/neuromuscular exercise OR Type 2—Mind–body Exercise including Tai Chi or Yoga) with or without Dietary Weight Management Topical NSAIDs
Conditionally recommended	Heat, Therapeutic cooling Cognitive behavioral therapy Acupuncture Kinesiotaping Balance training Patellofemoral knee brace Yoga Acetaminophen Tramadol Duloxetine Topical capsaicin	Aquatic exercise Gait aids Self-management programs Cognitive behavioral therapy with exercise Non-selective NSAIDs with or without PPIs (excluding patients with GI or CV comorbidities) COX-2 inhibitors (excluding patients with CV comorbidities) Intraarticular CSs Intraarticular hyaluronic acid

COX-2—cyclooxygenase-2; CSs—corticosteroids; CV—cardiovascular; GI—gastrointestinal; NSAIDs—non-steroidal anti-inflammatory drugs; PPIs—proton pump inhibitors.

## Data Availability

No new data were created or analyzed in this study. Data sharing is not applicable to this article.
